# Transcutaneous auricular vagal nerve stimulation modulates blood glucose in ZDF rats via intestinal melatonin receptors and melatonin secretion

**DOI:** 10.3389/fnins.2024.1471387

**Published:** 2024-11-05

**Authors:** Yuzhengheng Zhang, Ningyi Zou, Chen Xin, Yifei Wang, Zixuan Zhang, Peijing Rong, Shaoyuan Li

**Affiliations:** ^1^Key Laboratory of Ministry of Education for TCM Viscera-State Theory and Applications, Liaoning University of Traditional Chinese Medicine, Shenyang, Liaoning, China; ^2^Institute of Acupuncture and Moxibustion, China Academy of Chinese Medical Sciences, Beijing, China; ^3^Institute of Basic Research in Clinical Medicine, China Academy of Chinese Medical Sciences, Beijing, China

**Keywords:** transcutaneous auricular vagus nerve stimulation, melatonin, intestinal melatonin receptors, hypoglycemic effect, vagus nerve

## Abstract

**Background:**

Melatonin (MLT) and its receptor deficiency have been shown to be associated with type 2 diabetes mellitus (T2DM). Transcutaneous auricular vagus nerve stimulation (taVNS) is a non-invasive alternative intervention for patients suffering from hyperglycemia. Here, we aimed to investigate the role of taVNS on blood glucose modulation via intestinal melatonin receptors (MRs) and MLT secretion in hyperglycemia.

**Methods:**

Adult male Zucker diabetes fatty (ZDF) rats and Zucker lean (ZL) littermates were used. Forty ZDF rats were randomized into ZDF, taVNS, Px + taVNS and Lu + Px + taVNS groups (Px: pinealectomy, Lu: Luzindole). ZL rats served as a control group for comparison with ZDF rats without involvement in the taVNS intervention. Thirty min-taVNS interventions (2/15 Hz, 2 mA, 30 min/days) were administered once daily under anesthesia for 3 consecutive weeks in taVNS, Px + taVNS and Lu + Px + taVNS groups. Body weight and fasting blood glucose (FBG) were measured weekly in all rats, and real-time blood glucose was tested in the ZL and ZDF groups before, during and after the taVNS intervention. Plasma MLT concentration and the expression of MRs in the duodenum, jejunum and ileum were measured by the end of experiments.

**Results:**

Compared with the ZL group, the level of FBG and body weight increased (all *p* < 0.01), plasma MLT secretion and the expression of MRs in duodenum, jejunum and ileum of ZDF rats decreased obviously (all *p* < 0.05), respectively. TaVNS can significantly reverse the hyperglycemia by regulating the non-pineal-derived MLT and MRs system in Px + taVNS group. Compared with the ZDF group, the expression of different intestinal MRs in the taVNS group was increased and more compactly arranged (both *p* < 0.05), the level of plasma MLT secretion was up-regulated (*p* < 0.01), and FBG and body weight were decreased (both *p* < 0.01). Meanwhile, after taVNS intervention in rats in the Px + taVNS group, we observed an increase in MLT secretion and the number of intestinal MRs compared with the taVNS group (all *p* > 0.05). In contrast, ZDF rats in which the pineal gland was excised by taVNS intervention and injected with the MRs antagonist Luzindole did not show these changes.

**Conclusion:**

The glucose reduction effect of taVNS may be related to regulating MLT levels and expressing intestinal MRs.

## 1 Introduction

It is reported that melatonin (MLT) plays a protective role against type 2 diabetes mellitus (T2DM) through regulation of glucose metabolism in animals and patients via changes in insulin secretion and leptin production (Frese et al., 2009; Agil et al., 2012). Melatonin receptors (MRs) are guanosine-binding protein-coupled receptors and have now been found to be directly related to diabetes (Ventriglia et al., 2015). Epidemiological studies have reported an association between circadian rhythms and T2DM (Rizza et al., 2021). MLT and its receptors act as mediators in synchronizing rhythms with environmental rhythms in organisms (Stenvers et al., 2019). In this case, finding and activating a peripheral source of MLT secretion unaffected by circadian rhythms can improve the indices of T2DM.

Currently, the majority of drugs used for treating T2DM often lead to adverse effects such as hypoglycemia, gastrointestinal intolerance, and vitamin B12 deficiency (Fathallah et al., 2015). It is crucial to explore the development of more efficient non-pharmacological treatments, such as bioelectronic medicine, to manage the condition effectively. In the rat model, the auricular vagus nerve branch is one of the major nerve distributions in the ear, located mainly in the eardrum cavity (Peuker and Filler, 2002).

Although previous studies (Li et al., 2014) have found that transcutaneous auricular vagus nerve stimulation (taVNS) exerts a significant hypoglycaemic effect by ameliorating abnormal peripheral plasma MLT levels and that this effect is independent of pineal rhythmicity, these studies have focused solely on peripheral plasma MLT levels and have lacked a focus on specific organs and tissues, in particular the gut. This is important because of the significant advantages of melatonin in the gut, including its high production in the gut and its ability to regulate local and systemic metabolic processes (Garaulet et al., 2020). Studies have shown that melatonin in the gut may influence glucose metabolism and play an important role in insulin sensitivity and energy homeostasis (Yasmin et al., 2021). Furthermore, vagal control of gastrointestinal function is key in regulating gut-derived MLT (Reiter et al., 2003). Therefore, exploring the role of intestinal melatonin and its receptors in T2DM is crucial for a comprehensive understanding of the mechanisms of taVNS.

Still, the source of exogenous pineal MLT induced by taVNS is unknown. Notably, few studies have addressed intestinal MRs and MLT release in the pathological state of T2DM. Therefore, the present study explored the potential mechanisms by which taVNS stimulates peripheral MLT secretion by observing changes in intestinal MRs and MLT secretion in a rat model of T2DM under pathological conditions. This study design included assessments of taVNS intervention, pinealectomy (Px), and Luzindole (Lu, an MRs antagonist) post-injection to gain insight into the effects of these interventions. To accurately capture immediate and long-term physiological changes, we used a DSI telemetry system for dynamic monitoring of blood glucose in rats. This system allows us to monitor rats’ blood glucose levels in real-time while in a free-ranging state, thus assessing the changes in blood glucose associated with the intervention. The advantages of this technique are its non-invasiveness and minimal disturbance to the animal, allowing us to obtain more realistic physiological data under natural behavioral conditions. By combining real-time blood glucose monitoring and analysis of intestinal MRs and MLT secretion, the present study aims to provide insight into the role of taVNS in regulating blood glucose and its metabolism-related mechanisms.

## 2 Materials and methods

### 2.1 Animals

Male Zucker diabetic fatty (ZDF, 150 ± 15 g, *n* = 40) rats and Zucker lean (ZL, 130 ± 10 g, *n* = 10) rats, 6-week-old, were obtained from the Beijing Vital River Laboratory Animal Technology Co., Ltd. [License No. SCXK (Beijing) 2021–0006]. All rats were housed at the Institute of Acupuncture and Moxibustion, China Academy of Chinese Medical Sciences (CACMS). They were kept under controlled temperature (21°C ± 2°C), relative humidity (50% ± 10%) and in quiet environments with a 12-h light/dark cycle. Food and water were provided *ad libitum* to all rats except when indicated otherwise.

After 1 week of acclimatization, 10 ZL rats were selected into the ZL group. They were fed normal rat maintenance feed #1022. In addition, 40 ZDF rats were divided into four groups – ZDF, taVNS, Px + taVNS and Lu + Px + taVNS group, with 10 rats in each group. They were fed with high-fat feed, Purina #5008, which had 32% fat, 13% protein and 55% carbohydrates. To avoid any possible confounding effect from gender differences on the endogenous MLT level and other possible hormone variations, only male ZDF rats were used. All procedures were reviewed and approved by the Ethics Committee of the Institute of Acupuncture and Moxibustion, CACMS (NO. D2021-11-12-1).

### 2.2 Modeling

**Figure 1:**
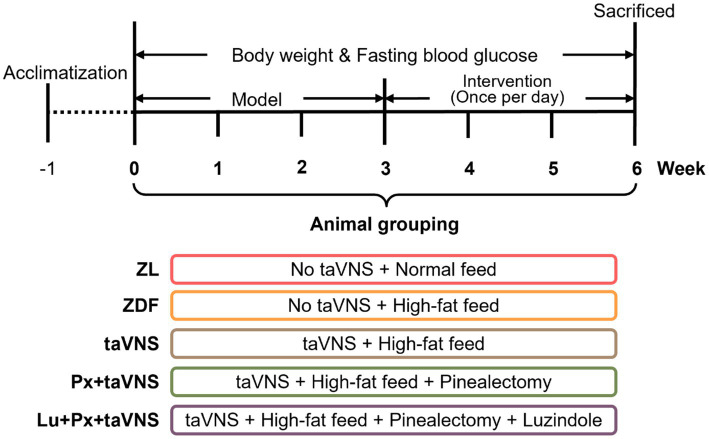
Experimental procedure and grouping. taVNS, transcutaneous auricular vagus nerve stimulation.

The ZDF rats were fed a customized high-fat Purina#5008 diet to create an elevated blood glucose-like model. The fasting blood glucose (FBG) concentration was measured weekly from tail tip blood using a glucometer. An FBG level of ≥11.1 mmol/L was used to determine the successful creation of the diabetes-like model (Yokoi et al., 2013). The test range was 0.6–33.3 mmol/L. Any concentration over the testing limit was recorded as 33.3 mmol/L for statistical purposes. The step-by-step process and the groupings followed during the experiment were illustrated in Figure 1.

### 2.3 Body weight and FBG measurements

The body weight and FBG were measured weekly from week 0 to week 6. Rats were placed individually in a plastic carrier with a weight meter to measure body weight in grams. The FBG level was immediately measured using the Ascensia Breeze Blood Glucose Monitoring System (Newbury, United Kingdom) by collecting blood samples from the tail with a test range of 0.6–33.3 mmol/L (Figure 1).

### 2.4 Intervention

The taVNS intervention was started at week 3 (Figure 1). The rats were subjected to electrical stimulation interventions under isoflurane inhalation anesthesia (4% concentration of isoflurane combined with 99.5% oxygen, the rats were induced to complete short-acting general anesthesia by perfusion and subsequently moved to the subchannel, and anesthesia concentration was reduced to 2% for maintenance) (Hebei Nine Sent Pharmaceutical Co., Ltd., Hebei, China). Positive and negative self-absorbing conductive electromagnets were connected and immobilized bilaterally in the auricular nail cavities of the rats in a non-invasive manner, and the observation of slight fluttering of the external auricle demonstrated the triumphant arrival of the stimulus. The intervention procedure consisted of 30 min of taVNS in the afternoon (ZT5-8) every day for 3 consecutive weeks. The stimulation parameters were: intensity 2 mA, frequency 2/15 Hz, sparse and dense waves, 30 min/session. An electrical stimulator (HANS-100, Nanjing, China) was used. These stimulus parameters and sites had proven effective in our previous study (Li et al., 2014).

### 2.5 Implantable blood glucose telemetry operation

Rats were anaesthetized by inhalation of 4% isoflurane (Hebei Nine Sent Pharmaceutical Co., Ltd., Hebei, China), and a 4–5 cm incision was made in the midline of the abdomen. The intra-abdominal tissues were stripped to expose the abdominal aorta, allowing clear visualization. Subsequently, the peri-abdominal aortic vessel was isolated, and vascular flow was blocked by proximal occlusion sutures at the lower end of the arterial branch of the left renal vein and distal occlusion sutures at the upper end of the iliac arterial branch. Using a curved 25-gauge syringe needle as a chip introducer, the abdominal aorta was punctured 1–2 mm to the iliac bifurcation, the chip was inserted and deepened upstream, and the implanted subchip segment was inserted into the vasculature and then withdrawn from the needle. The aortic entry site was sealed using swab drying and Vetbond tissue adhesive (3 M™ Vetbond™ Tissue Adhesive 1469SB). Two 3 mm × 5 mm fiber tabs secured the transducer (DATA SCIENCES INTERNATIONAL, INC., Minnesota, United States). After closing the incision, rats were injected with carprofen analgesia (5 mg/kg) in the medial thigh and kept warm postoperatively until they regained consciousness. The implanted chip’s specific serial number was matched with the external receiver to achieve minute-by-minute ambulatory blood glucose monitoring (Richard and Robert, 2012).

### 2.6 Pinealectomy

The pineal gland was surgically removed using a previously reported method (Maganhin et al., 2009) to explore the level of gut-secreted MLT and its receptor expression. Animals were fixed on a stereotaxic apparatus, and an incision was made along the midline of the skull. The skull was opened at the amnion using a dental drill (0.5 cm outer diameter) to visualize the superior sagittal sinus directly. During the procedure, the anterior and posterior sagittal sinuses were ligated with a No.5–0 silk thread and the pineal gland beneath the sagittal sinus was excised with forceps and sutured.

### 2.7 Intraperitoneal injection and plasma collection

A dose of vehicle Luzindole (30 mg/kg) was given weekly in the afternoon in Lu + Px + taVNS group. Luzindole was purchased from Sigma Chemical Co. (St. Louis, United States) and dissolved in 5% ethanol saline (v/v) immediately before use. To analyze the concentration of plasma MLT, blood was obtained from the abdominal aorta of rats anaesthetized by intraperitoneal injection of sodium pentobarbital (40 mg/kg). The blood samples were centrifuged for 10 min at 1,000 rpm, and the plasma was collected and stored at −80°C until use.

### 2.8 Enzyme-linked immunosorbent assay

The plasma MLT concentration was measured using an ELISA kit (Lot# DZE30014, R&D System, Beijing, China) by Huanya Biomedicine Technology Co. Ltd. The analysis was performed with a microplate reader (Multiskan MK3, Thermo Scientific, Beijing, China) at a wavelength of 450 nm. The results were calculated based on the standard curve and reported in nanograms per liter (ng/L).

### 2.9 Immunofluorescence staining

The rats were injected intravenously with sodium pentobarbital at 40 mg/kg, followed by cardio-perfusion with 200 mL of saline and 400 mL of 4% paraformaldehyde in a 0.1 M phosphate buffer (PB). The ileum, duodenum and jejunum were removed, postfixed for 2 h, and stored in 30% sucrose in a 0.1 M PB in a cold room until they sank to the bottom. Tissues were mounted in an OCT compound and frozen on dry ice. The ileum, duodenum and jejunum were then sectioned to 30 μm thickness using a cryostat, mounted serially onto microscope slides, and stored at −80°C. Immunohistochemical staining was used to detect MRs (1:1000, rabbit polyclonal; Abbiotec, San Diego, CA, United States). The ileum, duodenum and jejunum sections were blocked with 1% goat serum in 0.3% Triton × 100 for 1 h at room temperature and then incubated overnight at 4°C with the primary antibody. The sections were then incubated for 1 h at room temperature with a corresponding Cy3-conjugated secondary antibody (1:200, Jackson Immuno Research, West Grove, PA, United States). The ileum, duodenum and jejunum sections were observed using a LEXT OLS4000 3D Laser Measuring Microscope (Olympus), captured using a digital camera, and processed with Adobe Photoshop.

### 2.10 Western blot

Rats were decapitated while under anesthesia. Samples from the ileum, duodenum and jejunum were collected separately and then homogenized in an SDS buffer with a mixture of proteinase inhibitors from Sigma. The protein samples were separated on an SDS-PAGE gel, which was a 4–15% gradient gel from Bio-Rad in Hercules, CA, United States, and then transferred to polyvinylidene difluoride filters from Millipore in Bedford, MA, United States. The filters were clogged with 3% milk and left overnight at 4°C. They were then treated with an MRs primary antibody (40 kD, rabbit polyclonal, 1:500, Millipore, Billerica, MA, United States) and an HRP-conjugated secondary antibody (1:10,000; Abcam, Cambridge, MA, United States) for 1 h at room temperature. The blots were visualized in ECL solution (NEN, Boston, MA, United States) for 1 min and exposed to hyperfilms (Amersham Biosciences) for 1–10 min. After that, the blots were incubated in a stripping buffer (67.5 mM Tris; pH, 6.8; 2% SDS; and 0.7% *β*-mercaptoethanol) for 30 min at 50°C and probed again with a polyclonal rabbit anti-β-actin antibody (1:20,000; Alpha Diagnostic International, San Antonio, TX, United States) as the loading control. The Western analysis was conducted in triplicate. The density and size of the bands were measured with a computer-assisted imaging analysis system and normalized against loading controls.

### 2.11 Statistical methods

The data from the experiment was analyzed using *SPSS 25.0* statistical software, and *GraphPad Prism 8.4.0* software was utilized for graphing. The measurement data followed a normal distribution and were presented as mean ± standard deviation. *One-way ANOVA* was used to compare between groups, and two-by-two comparisons were made using the *LSD* method if the variances were equal. The *Kruskal-Wallis* method with the rank sum test was used if the variances were not equal. Correlation analyses were performed using linear regression analysis. Statistical significance was considered at *p* < 0.05.

## 3 Results

### 3.1 Comparison of body weight and blood glucose at different time points

**Figure 2:**
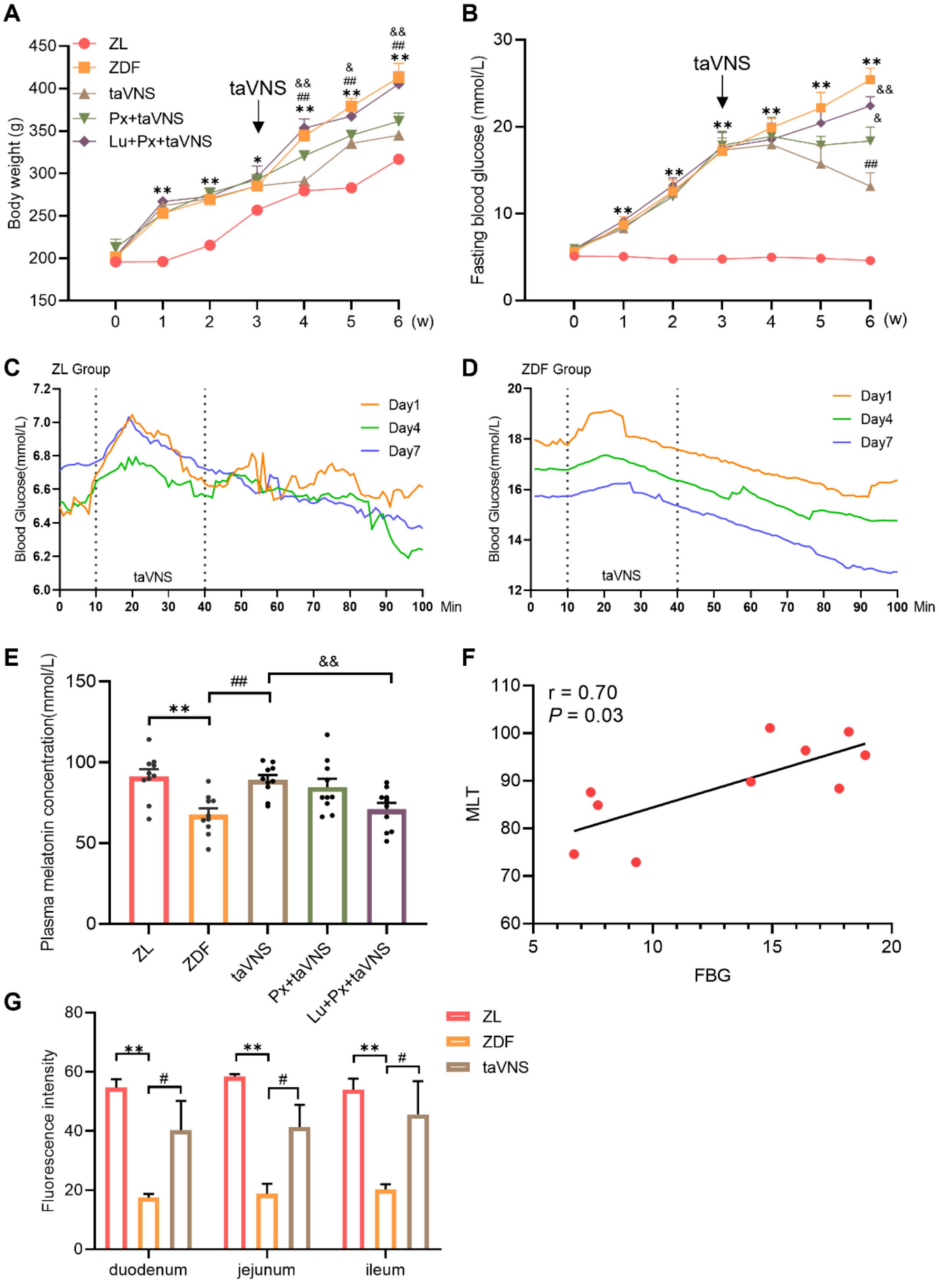
The effect of taVNS on the body weight, FBG, plasma MLT concentration, correlation analysis and IF of MRs from rat intestinal segments (*N* = 10). **(A)** Comparison of body weight at different time points. **(B)** Comparing the FBG at different time points. **(C,D)** Changes in blood glucose dynamics in ZDF rats before, during and after taVNS intervention. **(E)** Comparison of MLT concentration of rats. **(F)** Correlation analysis between MLT and FBG. **(G)** Cross-sectional comparison of immunofluorescence staining of MRs from rats’ duodenal, jejunal, and ileal sections. *p < 0.05 vs. ZL group, **p < 0.01 vs. ZL group; ^#^*p* < 0.05 vs. ZDF group, ^##^*p* < 0.01 vs. ZDF group; ^&^*p* < 0.05 vs. taVNS group, ^&&^*p* < 0.01 vs. taVNS group. *One-way ANOVA* followed by *Tukey’s* multiple comparison test. Data presented as mean ± SEM.

There was no significant difference in body weight among groups at baseline (0 week) (*p* > 0.05). The values in ZDF group were consistently higher than that of ZL group (*p* < 0.05 or *p* < 0.01). After taVNS intervention, the body weight value decreased continuously (*p* < 0.01) compared with ZDF group. Compared with taVNS group, the weight in Px + taVNS rats showed no significant changes (*p* > 0.05) but increased in Lu + Px + taVNS group (*p* < 0.05 or *p* < 0.01) (Figure 2A).

At week 0, there was no significant difference in FBG levels between different groups (*p* > 0.05). The ZDF rats experienced a gradual FBG increase (*p* < 0.01) compared with ZL group. After taVNS intervention, FBG values continuously decreased in taVNS group compared with ZDF group and reached the lowest point in week 6 (*p* < 0.01). Additionally, compared with taVNS group, the FBG in Px + taVNS group dropped but remained higher than taVNS group (*p* < 0.05), while Lu + Px + taVNS group showed a significant increase (*p* < 0.01) (Figure 2B).

Real-time blood glucose values were measured before, during and after the taVNS intervention. At 10–30 min of taVNS intervention, blood glucose in ZDF rats first increased and then decreased; at the same time point, the blood glucose value on day 7th was lower than that on day 1st and 4th, while the initial value of blood glucose on day 7th was lower than that on days 1st and 4th. The decrease in blood glucose still existed for 40–100 min after the cessation of taVNS intervention, and it gradually stabilized. The random blood glucose values of rats in ZL group gradually decreased to a stable level within 60 min at the end of the intervention, in which there was no statistical difference in blood glucose changes at 1–10 min versus 91–100 min on the first day, the fourth day and the seventh day (all *p* > 0.05); whereas, blood glucose in rats of the ZDF group showed fluctuations during the period of taVNS intervention, and gradually decreased after the intervention, in which there was a significant decrease in blood glucose from 1–10 min to 91–100 min on the first day, the fourth day, and the seventh day (all *p* < 0.001), and after the intervention lasting for 7 days, there was no significant difference in the blood glucose values of rats in the ZL group on the seventh day compared with that of rats on the first day (*p* > 0.05). There was a significant decrease in the blood glucose values of rats in the ZDF group (*p* < 0.001) (Figures 2C,D).

### 3.2 Effects of taVNS on plasma MLT concentration

Compared to ZL group, ZDF rats showed a significant reduction in plasma MLT concentration (*p* < 0.01). taVNS caused a reversal of MLT compared to the ZDF group (*p* < 0.01). Compared to the taVNS group, Px + taVNS group showed no significant difference (*p* > 0.05), while Lu + Px + taVNS group showed a significant reduction (*p* < 0.01). Otherwise, in ZDF rats, Spearman’s correlation analysis revealed a positive correlation between FBG and MLT (*r* = 0.7, *p* = 0.03) (Figures 2E,F).

### 3.3 Different expression of MRs in duodenum, jejunum, and ileum

**Figure 3:**
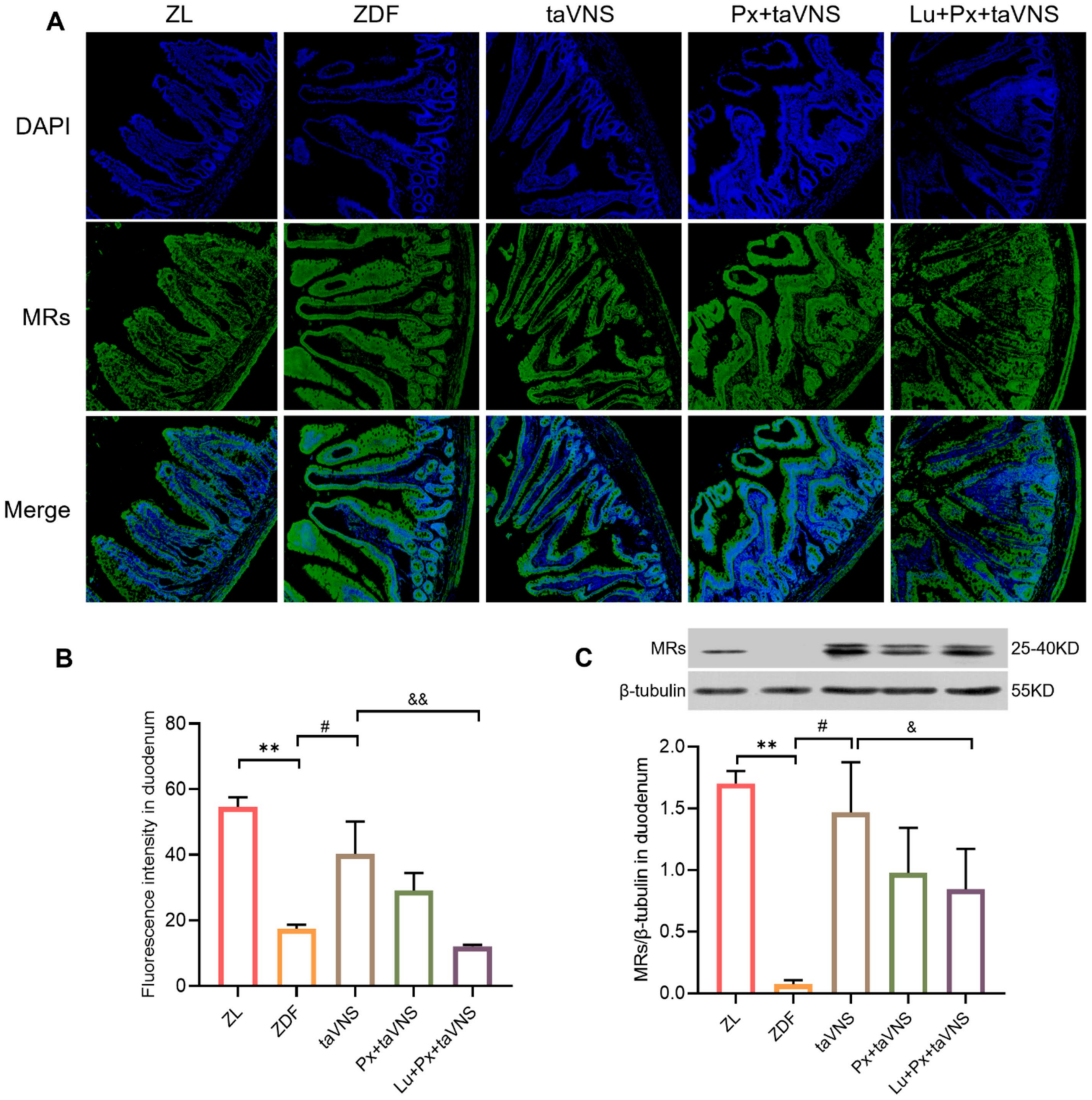
The expression of MRs in duodenum in different groups (*N* = 3). **(A)** MRs in duodenum (green), DAPI (blue); 40× objective, scale bar: magnification 200 μm. **(B)** The number of MRs in duodenal sections. **(C)** Comparison of MRs expression levels in the duodenum. **p < 0.01 vs. ZL group; ^#^*p* < 0.05 vs. ZDF group, ^##^*p* < 0.01 vs. ZDF group; ^&^*p* < 0.05 vs. taVNS group, ^&&^*p* < 0.01 vs. taVNS group. *One-way ANOVA* followed by *Tukey’s* multiple comparison test. Data presented as mean ± SEM.

**Figure 4:**
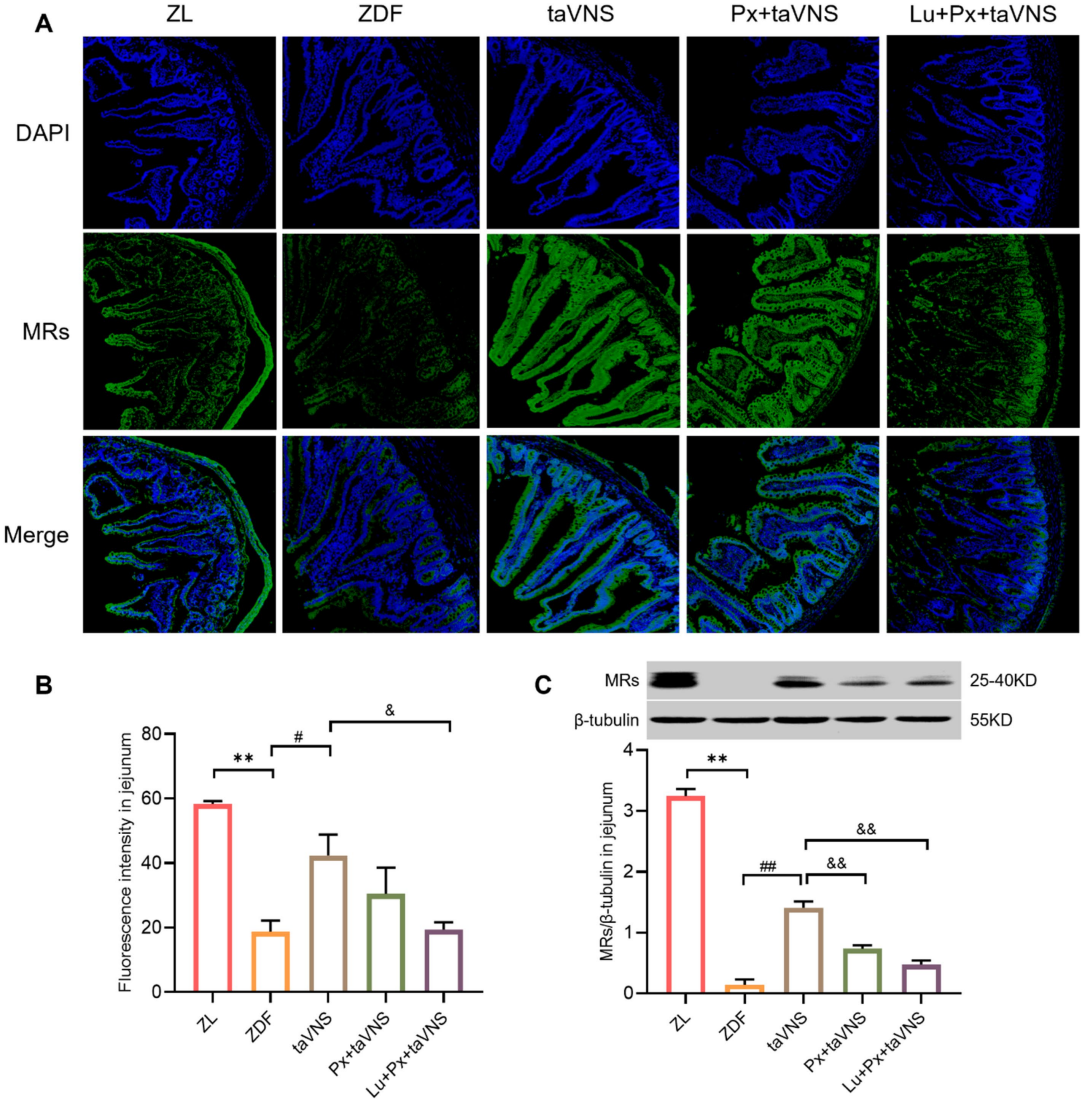
The expression of MRs in jejunum of different groups (*N* = 3). **(A)** MRs in jejunum (green), DAPI (blue); 40× objective, scale bar: magnification 200 μm. **(B)** The number of MRs in jejunal sections. **(C)** Comparison of MRs expression levels in the jejunum. **p < 0.01 vs. ZL group; ^#^*p* < 0.05 vs. ZDF group, ^##^*p* < 0.01 vs. ZDF group; ^&^*p* < 0.05 vs. taVNS group, ^&&^*p* < 0.01 vs. taVNS group. *One-way ANOVA* followed by *Tukey’s* multiple comparison test. Data presented as mean ± SEM.

**Figure 5:**
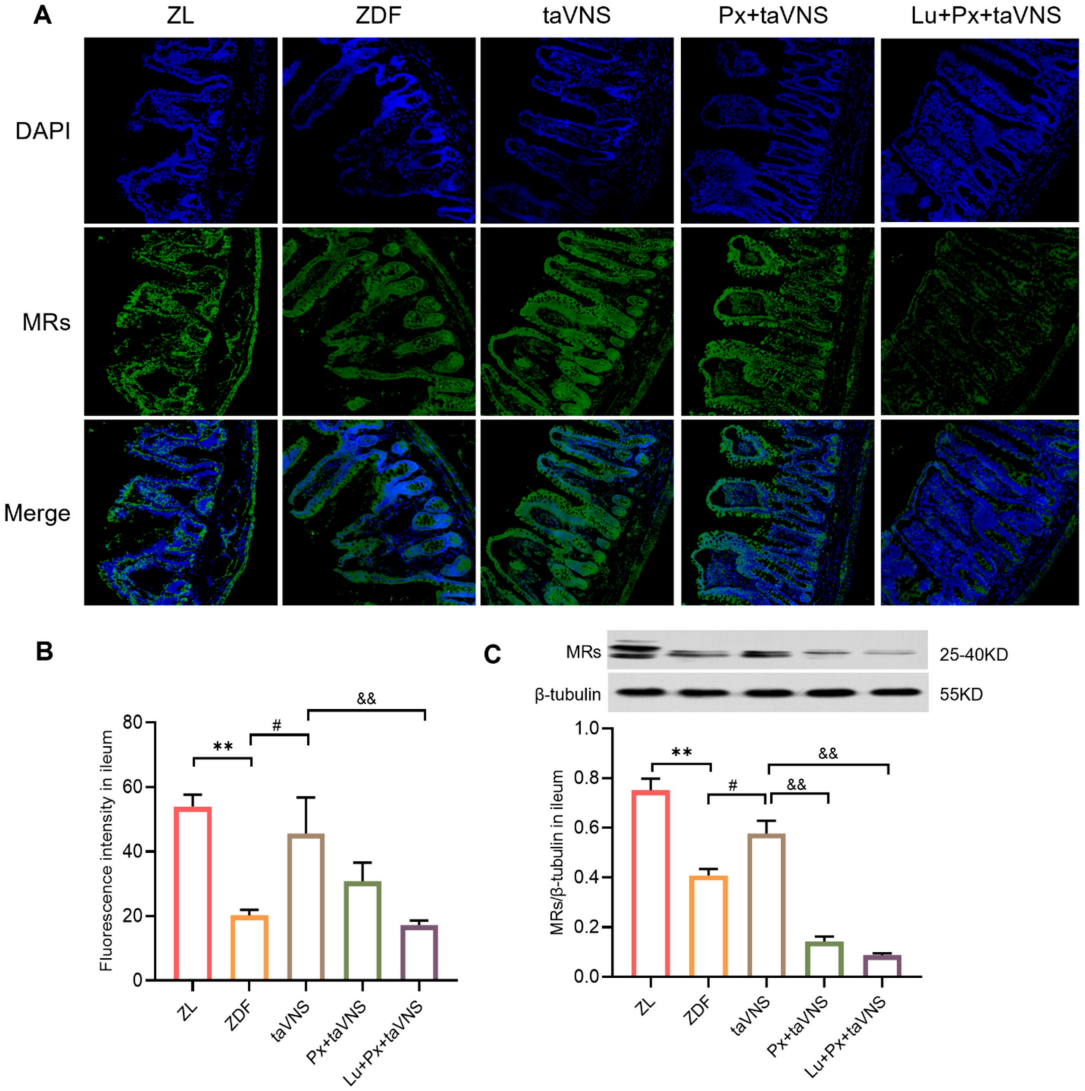
The expression of MRs in ileum of different groups (*N* = 3). **(A)** MRs in ileum (green), DAPI (blue); 40× objective, scale bar: magnification 200 μm. **(B)** The number of MRs in ileal sections. **(C)** Comparison of MRs expression levels in the ileum. **p < 0.01 vs. ZL group; ^#^*p* < 0.05 vs. ZDF group; ^&&^*p* < 0.01 vs. taVNS group. *One-way ANOVA* followed by *Tukey’s* multiple comparison test. Data presented as mean ± SEM.

It is worth noting that in Figure 2G, we made the side-by-side comparison of the number of MRs between different intestinal segments, which allows for a more intuitive view of the changes in the number of MRs in the intestinal segments in the disease state versus after the intervention. Compared to the ZL group, the number of MRs in the duodenum, jejunum, and ileum of ZDF group rats significantly decreased (*p* < 0.01). Among them, the duodenum exhibited a more significant reduction in MRs quantity, followed by a moderate decrease in the jejunum, while the ileum showed the slightest reduction in MRs. Morphologically, in comparison to ZL group, the MRs in different intestinal segments of the ZDF group displayed a lower density, with increased spacing between receptors, presenting a scattered distribution pattern (Figures 2G, 3A, 4A, 5A).

### 3.4 taVNS improves the distribution and upregulates the expression of MRs in the duodenum, jejunum, and ileum of ZDF rats

After intervention, compared to the ZDF group, the number of MRs in the duodenum, jejunum, and ileum of taVNS group increased significantly (all *p* < 0.05). The MRs exhibited a higher density distribution, with reduced spacing, presenting a tight and compact arrangement. Furthermore, compared to taVNS group, there was no significant difference in Px + taVNS group (all *p* > 0.05). However, the Lu + Px + taVNS group showed a decrease in MRs expression (*p* < 0.01, *p* < 0.05, *p* < 0.01) (Figures 3B, 4B, 5B).

Compared to ZDF group, taVNS group exhibited higher expression of MRs in all three intestinal segments (*p* < 0.05, *p* < 0.01, *p* < 0.05). When compared to taVNS group, both Px + taVNS group and Lu + Px + taVNS group showed a significant decrease in MRs expression in jejunum and ileum (both *p* < 0.01). In duodenum, Lu + Px + taVNS group demonstrated a decrease in MRs expression (*p* < 0.05), while Px + taVNS group showed no statistically significant difference (*p* > 0.05) (Figures 3C, 4C, 5C).

## 4 Discussion

T2DM is a metabolic disease that affects multiple organs. Recent findings indicate that MLT and its receptors are crucial in T2DM. In the pathological state of T2DM, the pineal gland, the main central organ for MLT secretion, exhibits pathological changes such as a decrease in precursor or cells for MLT synthesis, abnormal expression of MLT synthetic enzyme system, and even the risk of accelerated peripheral neuropathy (Song et al., 2017). How to activate other peripheric organs is imperative to enhance MLT secretion. Our previous studies (Wang et al., 2015) have observed that taVNS intervention elevated plasma MLT levels in pinealectomized rats. This led us to infer that taVNS could stimulate MLT secretion in organs beyond the central pineal gland in ZDF rats. It is reported that the MLT content in gastrointestinal tract is 400 times that of pineal gland, which is the “largest MLT storage pool” in the human body (Bubenik, 2002). Gastrointestinal MLT secretion is not controlled by circadian rhythm, and the number of MLT-producing enterochromaffin cells has been observed to show a compensatory increase after removing the pineal gland. Therefore, based on the relationship between T2DM and plasma MLT content and the number of different intestinal MRs, the present study innovatively explored the mechanism of action of taVNS from morphology and molecular biology and elucidated that taVNS improves the abnormalities of glycaemic metabolism in ZDF rats by modulating the number of intestinal MLT and its receptors.

The ZDF rat is a T2DM-like animal model that develops naturally after a high-fat diet. This leads to glucose intolerance, insulin resistance, and damage to pancreatic *β*-cells. It is commonly used in the research of T2DM and its complications. The ZL rat is used as a control group for its homologous line and is mainly utilized for comparative studies of T2DM mechanisms (Yokoi et al., 2013).

The MLT is an endogenously produced indole hormone that has been scientifically shown to have the ability to reduce abnormally elevated blood glucose and lipids. This, in turn, can mitigate the harmful effects of glucolipotoxicity on pancreatic β-cells (Agil et al., 2012). MLT possesses antioxidant and anti-inflammatory properties that can effectively lower the level of oxidative stress in the pancreas (Kaneto et al., 2010). As a result, MLT can play a crucial role in protecting β-cells and improving the process of T2DM. After intraperitoneal injection of MLT, FBG, glycosylated hemoglobin, and Fructosamine are reduced to varying degrees, resulting in improved glucose metabolism in diabetic mice (Shieh et al., 2009). After MLT is secreted from intestinal chromaffin cells (Yasmin et al., 2021), it regulates glucose metabolism by altering insulin receptor dependence. It reduces the concentration of insulin antagonist hormones, such as cortisol, in the blood, improving glucose metabolism and insulin resistance. MLT released from the intestine is not dependent on the circadian cycle. It acts directly on regulating blood glucose in the organism, which explains that in pinealectomized rats, a decrease in body weight and blood glucose and increased plasma MLT levels were still observed. These results suggest intestinal MLT secretion has a lasting regulatory effect on body weight and blood glucose.

As typical G-protein coupled receptors, MRs are usually expressed independently at the plasma membrane as monomeric receptors (Patke et al., 2020). When MRs are coupled to Gq, they activate phosphodiesterase C and increase insulin secretion (Choi et al., 2014; Jenwitheesuk et al., 2014). In the absence of the MRs in mice, glucose metabolism is reduced. It is hypothesized that the activation of the MRs can promote glucose metabolism, improving insulin resistance and maintaining glucose homeostasis (Zephy and Ahmad, 2015). Genetic variation in the MRs gene increases the risk of T2DM, according to genome-wide association studies (Ventriglia et al., 2015). The expression of MRs varied by the different tissues. In rats, the level of MRs mRNA was the highest in the duodenum and colon (Sallinen et al., 2005). The expression of MRs was observed in various parts of the human gastrointestinal tract, including the intestinal epithelium, submucosa, intermuscular plexus, and gastrointestinal vasculature. The colorectal epithelium showed the highest expression level.

Luzindole acts as an antagonist of MRs receptors, and ZDF rats exhibited elevated blood glucose, decreased serum MLT levels, and reduced receptor expression in the jejunum, duodenum and ileum after injection of the antagonist in this experiment, demonstrating that taVNS affects MLT secretion by regulating the number of intestinal MRs, thereby improving T2DM.

The present study investigated the impact of high-fat chow feeding on blood glucose levels in ZDF rats (Li et al., 2014). In this experiment, taVNS was given to ZL and ZDF rats in the awakened state through the DSI experimental animal physiological signal telemetry system to observe the intervention’s immediate effect on blood glucose. A rise in blood glucose concentration was observed in the initial phase of the intervention, probably due to the activation of the pancreatic *α*-cells, increasing the release of glucagon; however, this rise was transient, which agreed with the results of previous experiments. These results are consistent with previous experiments, supporting the reversal effect of taVNS on hyperglycemia (Zhang et al., 2021). The study results indicate that taVNS reversed the observed phenomenon of elevated blood glucose levels, thus pointing toward its potential as a therapeutic intervention to lower blood glucose levels. taVNS has been found to produce hypoglycemic effects in ZDF rats. This is achieved by increasing the number of MRs in the jejunum, duodenum, and ileum, promoting intestinal MLT secretion. It has also been shown that gastrointestinal MRs play a role in regulating blood glucose metabolism. *In vivo*, studies have demonstrated that after the injection of an MRs antagonist, both blood glucose and plasma MTL content decreased in ZDF rats. Based on these findings, we hypothesize that gastrointestinal MRs regulate MTL content, thus contributing to the development of T2DM. The auricular area has a visceral region in the area of the auricular branch of the vagus nerve, the only region on the surface of mammals with this type of nerve distribution. When electrical stimulation is applied to the vagus nerve endings in this region, the electrical impulses travel through the pseudo-unipolar neuron to the solitary tract nucleus and then connect with the dorsal nucleus of the vagus nerve. This results in the activation of the parasympathetic centers of the brainstem and the limbic system (Peuker and Filler, 2002). Bipolar neurons transmit electrical impulses from the hypothalamus to the target organs, such as the pancreas (Bloom and Edwards, 1985). These specialized neurons utilize longer nerve fibers to reach these organs’ parasympathetic and intramural ganglia, ultimately facilitating the innervation of peripheral target organs post-metamorphosis (Berthoud et al., 1990). The previous experiments showed that taVNS significantly elevated serum insulin concentration in pinealectomized ZDF rats, improved the status of high glycosylated hemoglobin levels and significantly lowered blood glucose. The effect was similar to the efficacy of an intraperitoneal injection of MLT (Wang et al., 2015).

Transcutaneous auricular vagus nerve stimulation, a non-invasive neuromodulation technique, demonstrates potential clinical application value in treating T2DM. The results of this study indicate that taVNS regulates intestinal melatonin and its receptor system, effectively lowering blood glucose levels and potentially positively influencing insulin sensitivity and overall glucose metabolism. Therefore, taVNS holds promise as an adjunctive therapy for T2DM, particularly for patients with limited response to medication or who experience side effects, offering a safe and feasible alternative.

Furthermore, taVNS’s non-invasive nature and relatively simple procedure provide a solid foundation for its promotion in clinical practice. Future clinical studies should focus on taVNS’s effects on long-term blood glucose control and its potential protective effects against complications in T2DM patients, such as cardiovascular disease and diabetic neuropathy. These studies will provide crucial data for further optimizing the application parameters of taVNS and clinical treatment protocols.

## 5 Conclusion

Compared to their lean littermates, ZDF rats innately develop T2DM with reduced MLT and MRs between various intestinal segments. taVNS has a glucose-gain attenuating effect in ZDF rats that appears to be mediated by increased MLT secretion and MRs. It would be interesting to investigate the potential role of taVNS in combination with MLT secretion as an adjunctive intervention for T2DM.

## 6 Limitations

This study demonstrates the effect of taVNS in regulating blood glucose through intestinal MRs; however, several limitations remain. Although the current research highlights the regulatory effects of taVNS, the specific molecular mechanisms have not been fully elucidated. Additionally, we observed a positive correlation between melatonin secretion and blood glucose levels, but the precise regulatory mechanisms require further investigation. To address this gap, we plan to incorporate more refined molecular tools, such as siRNA or CRISPR-Cas9 gene knockdown techniques, in future experiments to explore the roles of relevant signaling pathways. This approach will enhance our understanding of how taVNS influences insulin sensitivity and glucose metabolism through biological signaling networks.

While we have presented initial data on the impact of taVNS on blood glucose regulation and insulin sensitivity, we intend to include functional assessments, such as glucose tolerance tests and insulin sensitivity tests, in future studies to thoroughly validate the role of taVNS in metabolic regulation.

## Data Availability

The datasets presented in this article are not readily available because the data that support the findings of this study are available on request from the corresponding author, upon reasonable request. Requests to access the datasets should be directed to Shaoyuan Li, 704488328@qq.com.

## References

[ref1] Agil A. Rosado I. Ruiz R. Figueroa A. Zen N. Fernández-Vázquez G. (2012). Melatonin improves glucose homeostasis in Young Zucker diabetic fatty rats. J. Pineal Res. 52, 203–210. doi: 10.1111/j.1600-079X.2011.00928.x, PMID: 21883445

[ref2] Berthoud H. R. Jedrzejewska A. Powley T. L. (1990). Simultaneous labeling of vagal innervation of the gut and afferent projections from the visceral forebrain with Dil injected into the dorsal vagal complex in the rat. J. Comp. Neurol. 301, 65–79. doi: 10.1002/cne.903010107, PMID: 1706359

[ref3] Bloom S. R. Edwards A. V. (1985). Effects of certain metabolites on pancreatic endocrine responses to stimulation of the Vagus nerves in conscious calves. J. Physiol. 362, 303–310. doi: 10.1113/jphysiol.1985.sp015678, PMID: 3894623 PMC1192897

[ref4] Bubenik G. A. (2002). Gastrointestinal melatonin: localization, function, and clinical relevance. Dig. Dis. Sci. 47, 2336–2348. doi: 10.1023/a:1020107915919, PMID: 12395907

[ref5] Choi T. Y. Kwon J. E. Durrance E. S. Jo S. H. Choi S. Y. Kim K. T. (2014). Melatonin inhibits voltage-sensitive ca(2+) channel-mediated neurotransmitter release. Brain Res. 1557, 34–42. doi: 10.1016/j.brainres.2014.02.023, PMID: 24560601

[ref6] Fathallah N. Slim R. Larif S. Hmouda H. Ben S. C. (2015). Drug-induced Hypergl-ycaemia and diabetes. Drug Saf. 38, 1153–1168. doi: 10.1007/s40264-015-0339-z 26370106

[ref7] Frese T. Bach A. G. Mühlbauer E. Pönicke K. Brömme H. J. Welp A.. (2009). Pineal melatonin synthesis is decreased in type 2 diabetic Goto-Kakizaki rats. Life Sci. 85, 526–533. doi: 10.1016/j.lfs.2009.08.004, PMID: 19695268

[ref8] Garaulet M. Qian J. Florez J. C. Arendt J. Saxena R. Scheer F. A. J. L. (2020). Melatonin effects on glucose metabolism: time to unlock the controversy. Trends Endocrinol Metab 31, 192–204. doi: 10.1016/j.tem.2019.11.011, PMID: 31901302 PMC7349733

[ref9] Jenwitheesuk A. Nopparat C. Mukda S. Wongchitrat P. Govitrapong P. (2014). Melatonin regulates aging and neurodegeneration through energy metabolism, epigenetics, autophagy and circadian rhythm pathways. Int. J. Mol. Sci. 15, 16848–16884. doi: 10.3390/ijms150916848, PMID: 25247581 PMC4200827

[ref10] Kaneto H. Katakami N. Matsuhisa M. Matsuoka T. A. (2010). Role of reactive oxygen species in the progression of type 2 diabetes and atherosclerosis. Mediat. Inflamm. 2010:453892, 1–11. doi: 10.1155/2010/453892, PMID: 20182627 PMC2825658

[ref12] Li S. Zhai X. Rong P. McCabe M. F. Wang X. Zhao J.. (2014). Therapeutic effect of Vagus nerve stimulation on depressive-like behavior, hyperglycemia and insulin receptor expression in Zucker fatty rats. PLoS One 9:e112066. doi: 10.1371/journal.pone.0112066, PMID: 25365428 PMC4218831

[ref13] Li S. Zhai X. Rong P. McCabe M. F. Zhao J. Ben H.. (2014). Transcutaneous auricular Vagus nerve stimulation triggers melatonin secretion and is antidepressive in Zucker diabetic fatty rats. PLoS One 9:e111100. doi: 10.1371/journal.pone.0111100, PMID: 25347185 PMC4210269

[ref14] Maganhin C. C. Simões R. S. Fuchs L. F. Oliveira-Filho R. M. MdeJ S. Evêncio Neto J.. (2009). Rat Pinealectomy: a modified direct visual approach. Acta Cir. Bras. 24, 321–324. doi: 10.1590/s0102-86502009000400013, PMID: 19705033

[ref15] Patke A. Young M. W. Axelrod S. (2020). Molecular mechanisms and physiological importance of circadian rhythms. Nat. Rev. Mol. Cell Biol. 21, 67–84. doi: 10.1038/s41580-019-0179-2 31768006

[ref16] Peuker E. T. Filler T. J. (2002). The nerve supply of the human auricle. Clin. Anat. 15, 35–37. doi: 10.1002/ca.1089 11835542

[ref17] Reiter R. J. Tan D. X. Mayo J. C. Sainz R. M. Leon J. Bandyopadhyay D. (2003). Neurally-mediated and neurally-independent beneficial actions of melatonin in the gastrointestinal tract. J. Physiol. Pharmacol. 54, 113–125. 15075454

[ref18] Richard G. P. Robert B. (2012). Assessment of Nova biomedical StatStrip^®^ glucose meters and test strips in rodent glucose studies. FASEB J. 26:1127.11. doi: 10.1096/fasebj.26.1_supplement.1127.11

[ref19] Rizza S. Luzi A. Mavilio M. Ballanti M. Massimi A. Porzio O.. (2021). Alterations in rev-ERBα/BMAL1 ratio and glycated hemoglobin in rotating shift workers: the EuRhythDia study. Acta Diabetol. 58, 1111–1117. doi: 10.1007/s00592-021-01676-z, PMID: 33788000 PMC8272695

[ref20] Sallinen P. Saarela S. Ilves M. Vakkuri O. Leppäluoto J. (2005). The expression of MT1 and MT2 melatonin receptor mRNA in several rat tissues. Life Sci. 76, 1123–1134. doi: 10.1016/j.lfs.2004.08.016, PMID: 15620576

[ref21] Shieh J. M. Wu H. T. Cheng K. C. Cheng J. T. (2009). Melatonin ameliorates high fat diet-induced diabetes and stimulates glycogen synthesis via a PKCzeta-Akt-GSK3beta pathway in hepatic cells. J. Pineal Res. 47, 339–344. doi: 10.1111/j.1600-079X.2009.00720.x, PMID: 19817973

[ref22] Song J. Whitcomb D. J. Kim B. C. (2017). The role of melatonin in the onset and progression of type 3 diabetes. Mol. Brain 10:35. doi: 10.1186/s13041-017-0315-x, PMID: 28764741 PMC5539639

[ref23] Stenvers D. J. Scheer F. Schrauwen P. la Fleur S. E. Kalsbeek A. (2019). Circadian clocks and insulin resistance. Nat. Rev. Endocrinol. 15, 75–89. doi: 10.1038/s41574-018-0122-1 30531917

[ref24] Ventriglia G. Nigi L. Sebastiani G. Dotta F. (2015). MicroRNAs: novel players in the dialogue between pancreatic islets and immune system in autoimmune diabetes. Biomed. Res. Int. 2015:749734. doi: 10.1155/2015/749734, PMID: 26339637 PMC4538424

[ref25] Wang S. Zhai X. Li S. McCabe M. F. Wang X. Rong P. (2015). Transcutaneous Vagus nerve stimulation induces tidal melatonin secretion and has an antidiabetic effect in Zucker fatty rats. PLoS One 10:e0124195. doi: 10.1371/journal.pone.0124195, PMID: 25880500 PMC4400163

[ref26] Yasmin F. Sutradhar S. Das P. Mukherjee S. (2021). Gut melatonin: a potent candidate in the diversified journey of melatonin research. Gen. Comp. Endocrinol. 303:113693. doi: 10.1016/j.ygcen.2020.113693, PMID: 33309697

[ref27] Yokoi N. Hoshino M. Hidaka S. Yoshida E. Beppu M. Hoshikawa R.. (2013). A novel rat model of type 2 diabetes: the Zucker fatty diabetes mellitus ZFDM rat. J. Diabetes Res. 2013:103731. doi: 10.1155/2013/103731, PMID: 23671847 PMC3647587

[ref28] Zephy D. Ahmad J. (2015). Type 2 diabetes mellitus: role of melatonin and oxidative stress. Diabetes Metab. Syndr. 9, 127–131. doi: 10.1016/j.dsx.2014.09.018, PMID: 25450812

[ref29] Zhang Z. X. Li S. Y. Wang Y. Zhang Y. Wang Y. F. Rong P. J. (2021). Effect of transcutaneous auricular Vagus nerve stimulation on fasting blood glucose and serum insulin concentration in Zucker diabetes fatty rats. World J. Acupunct. Mox. 31, 212–217. doi: 10.1016/j.wjam.2021.05.002

